# Pyrido[2,3-*d*]pyrimidin-7(8*H*)-ones: Synthesis and Biomedical Applications

**DOI:** 10.3390/molecules24224161

**Published:** 2019-11-16

**Authors:** Guillem Jubete, Raimon Puig de la Bellacasa, Roger Estrada-Tejedor, Jordi Teixidó, José I. Borrell

**Affiliations:** Grup de Química Farmacèutica, IQS School of Engineering, Universitat Ramon Llull, Via Augusta 390, E-08017 Barcelona, Spain; guillemjubetea@iqs.edu (G.J.); raimon.puig@iqs.url.edu (R.P.d.l.B.); roger.estrada@iqs.url.edu (R.E.-T.); jordi.teixido@iqs.edu (J.T.)

**Keywords:** pyrido[2,3-*d*]pyrimidines, 5,6-dihydropyrido[2,3-*d*]pyrimidin-7(8*H*)-ones, biological activity, substitution pattern

## Abstract

Pyrido[2,3-*d*]pyrimidines (**1**) are a type of privileged heterocyclic scaffolds capable of providing ligands for several receptors in the body. Among such structures, our group and others have been particularly interested in pyrido[2,3-*d*]pyrimidine-7(8*H*)-ones (**2**) due to the similitude with nitrogen bases present in DNA and RNA. Currently there are more than 20,000 structures **2** described which correspond to around 2900 references (half of them being patents). Furthermore, the number of references containing compounds of general structure **2** have increased almost exponentially in the last 10 years. The present review covers the synthetic methods used for the synthesis of pyrido[2,3-*d*]pyrimidine-7(8*H*)-ones (**2**), both starting from a preformed pyrimidine ring or a pyridine ring, and the biomedical applications of such compounds.

## 1. Introduction

**Figure 1:**
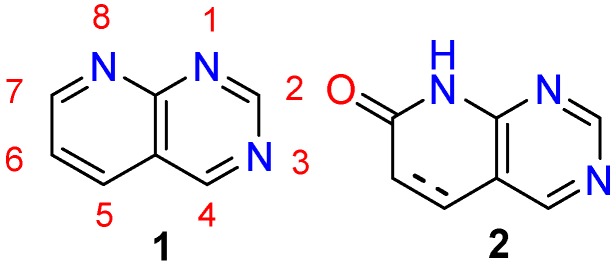
Structures of the pyrido[2,3-*d*]pyrimidine ring system (**1**) and pyrido[2,3-*d*]pyrimidin-7(8*H*)-ones (**2**).

Pyridopyrimidines are *ortho*-fused bicyclic heterocyclic structures formed by the fusion of a pyridine and a pyrimidine ring. There are four possible isomeric pyridopyrimidines [1,2] and one of them is pyrido[2,3-*d*]pyrimidines (1,3,8-triazanaphtalenes) **1** shown in Figure 1. Such structure is included in the concept of *privileged heterocyclic scaffolds*, introduced by Evans in the late 80s [3] and recently revised by Altomare [4], for drug discovery probably due to their resemblance with DNA bases. Some examples of drugs based on such structure that have reached the market are piritrexim isethionate (treatment of bladder cancer and urethral cancer) and pipemidic acid (antibiotic active against Gram-negative and some Gram-positive bacteria) [5].

**Figure 2:**
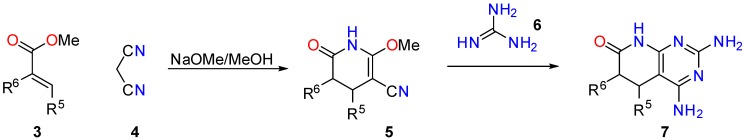
Synthesis of 2,4-diamino-5,6-dihydropyrido[2,3-*d*]pyrimidin-7(8*H*)-ones (**7**) from 2-methoxy-6-oxo-1,4,5,6-tetrahydropyridine-3-carbonitriles (**5**).

Among these bicyclic heterocyclic compounds, our group has been especially interested in the pyrido[2,3-*d*]pyrimidin-7(8*H*)-ones (**2**) (Figure 1) since the initial synthetic approach of Victory et al. to 2,4-diamino-5,6-dihydropyrido[2,3-*d*]pyrimidin-7(8*H*)-ones (**7**) obtained by reaction of 2-methoxy-6-oxo-1,4,5,6-tetrahydropyridine-3-carbonitriles (**5**), prepared upon treatment of an α,β-unsaturated ester (**3**) with malononitrile (**4**) in the presence of NaOMe/MeOH, and guanidine (**6**) (Figure 2) [6]. Since then, our group has developed synthetic methodologies allowing to access systems **2** with up to five diversity centers (C2, C4, C5, C6, and N8) and two degrees of unsaturation C5-C6 (note: during the rest of the review substituents at positions C2, C4, C5, C6, and N8 will be depicted as G_2_, G_4_, R_5_, R_6_ and R_8_ for a better comparison of the structures).

A preliminary search carried out in *SciFinder* [7] has revealed that there are more than 20,000 compounds which include the substructure **2** (both with a C5-C6 single and double bond) and more than 2900 references. Moreover, the number of references including such substructure has almost exponentially increased during the past 10 years showing the interest for such kind of systems and their potential biomedical applications.

**Figure 3:**
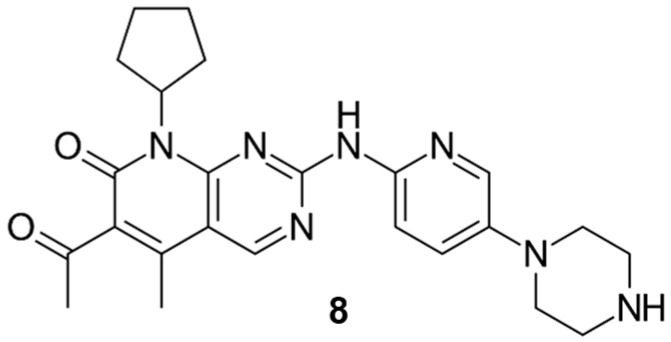
Structure of palbociclib (**8**) approved for the treatment of breast cancer.

However, if the total number of references is filtered to select the Reviews, the number is reduced to around 180. An inspection of such references indicates that most of them are reviews on the biological activity of pyrido[2,3-*d*]pyrimidin-7(8*H*)-ones (**2**) (mainly as kinase inhibitors [8,9], anti-leukemic [10], against breast cancer [11], antihypertensives [12], etc.) or about the compound palbociclib (**8**) approved for the treatment of breast cancer (Figure 3) [13].

The lack of a specific revision on pyrido[2,3-*d*]pyrimidin-7(8*H*)-ones (**2**) impelled us to review the literature covering structural, synthetic, and biological aspects.

## 2. Structural Features of Pyrido[2,3-*d*]pyrimidin-7(8*H*)-ones: Substitution Patterns and Degree of Unsaturation C5-C6

One of the interesting aspects to be included in a review of a given substructure is to have an idea of the variety of substituents that have been introduced in each diversity center of the molecule. This information is useful to know the diversity already covered in a Markush formula based on such substructure, particularly when a new project related to it has to begin looking for new biological activities and a patent. Before the introduction of computerized databases, such as *SciFinder*, this information was virtually inaccessible by requiring the review of hundreds of articles.

**Figure 4:**
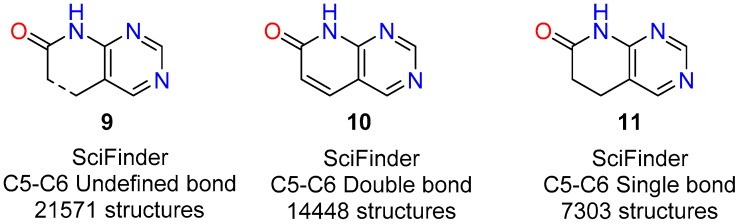
Number of pyrido[2,3-*d*]pyrimidin-7(8*H*)-ones (**2**) retrieved using an *undefined bond* (**9**), double bond (**10**) and single bond (**11**) between C5 and C6, respectively.

To carry out such analysis for the pyrido[2,3-*d*]pyrimidin-7(8*H*)-ones (**2**), the first step was to determine the total number of structures **2** present in the database. For this purpose, we used in the substructural search the *Unspecified bond* tool between C5 and C6 (depicted by a discontinuous line in **9**, Figure 4) that includes single, double, or triple bonds between those positions. The search gave a total number of 21,571 pyrido[2,3-*d*]pyrimidin-7(8*H*)-ones (**2**), a number that is slowly but continuously growing (search carried out in May 2019). Then we carried out the searches with a C5-C6 double bond **10** (14,448 structures) and a C5-C6 single bond **11** (9183 structures) (Figure 4). A quick initial search related to the single bond retrieved 9183 results but, surprisingly, it included some double-bonded structures, so it was curated by means of the *Exclude* command of the *Combine Answer Sets* tool in *SciFinder* to obtain the final set of 7303 molecules. It is interesting to note that from the total number of structures **2** included in *SciFinder* roughly 2/3 (67%) correspond to structures presenting a C5-C6 double bond. Such a 2:1 ratio in favor of the C5-C6 double bond can be due both to a structural requirement for the biological activity of compounds **2** (mostly used as tyrosine kinase inhibitors as described later) or to the easier synthetic approaches for such unsaturated structures also described later.

The structures **10** presenting a C5-C6 double bond are contained in around 2500 references which include around 1100 patents (43.6%) showing the great interest of such kind of structures. On the other hand, the structures **11** with a C5-C6 single bond appear in less than 500 references (a number clearly lower than the preceding one) although 60% are patents.

The huge number of compounds retrieved makes it impossible to download the structures from the database to perform a diversity analysis with specialized software (*SciFinder* allows the creation of an SDFile with the structures retrieved on a search but it is limited to 500 compounds). Consequently, we decided to explore one by one the substitution patterns at positions C2, C4, C5, C6, and N8 for each degree of unsaturation C5-C6 to have a picture of the diversity covered by the substances already described.

### 2.1. Substitution Pattern at C2 and C4

Concerning positions C2 and C4 we searched the structures presenting H, C (either alkyl groups or aromatic rings), N (primary amines, aminoalkyl or aminoaryl groups or heterocyclic rings connected by the nitrogen atom), O (hydroxy group probably as the carbonyl tautomer, ethers or ester groups), and S (thiol groups, thioethers, or SO_2_Me groups used for the subsequent nucleophilic substitution) as possible substituents both for the C5-C6 single and double bonds. The results obtained are included in Table 1 and Table 2, respectively, which include also examples of references containing such substitution patterns.

Concerning 5,6-dihydropyrido[2,3-*d*]pyrimidin-7(8*H*)-ones (**11**) (Table 1), the inspection of the percentages reveals that the nitrogen-based substituents at position C2 are the majority (more than 43%) followed by the carbon substituents (around 30%) and sulfur substituents (21%). On the other hand, the oxygen substituents, particularly as a carbonyl group, prevailed at position C4 (around 63%) followed also by the carbon substituents (almost 26%). It is interesting to note that the added percentages at position C2 cover 99.63% of the diversity at such position while in the case of C4 reach 98.99%. We also carried out a search corresponding to the combination of nitrogen substituent at C2 and oxygen substituent at C4 which covers the 37.97% of the total diversity, but includes only around 30 references. Such percentage is high enough taking into account that it corresponds to one of the 20 combinations (5 × 4) possible and can be interpreted on the basis of the similarity of the resulting structures with guanine. In the case of the double nitrogen substitution at C2 and C4, the total number of structures covers 4.30% of the total diversity, the corresponding references coming from our group of research.

In the case of pyrido[2,3-*d*]pyrimidin-7(8*H*)-ones (**10**) with a C5-C6 double bond (Table 2), the analysis of the substitution pattern at C2 indicates the nitrogen substituents are absolutely predominant (near 76%), very far from the other possible substitutions. Similarly, in the case of C4, the presence of a hydrogen atom represents 78% of the situations followed by the carbon substituents (near 18%). Now the addition of the percentages of the different substituents considered reaches 89.34% and 99.64% for C2 and C4, respectively. The combination of a nitrogen substituent at C2 and a hydrogen atom at C4 covers the 63.72% of the total diversity, thus revealing that such combination has been largely explored in connection with the biological activity of these structures as antineoplastic drugs that will be discussed later. 

### 2.2. Substitution Pattern at C5 and C6

While the combination of substituents at C2 and C4 of pyrido[2,3-*d*]pyrimidin-7(8*H*)-ones (**2**) is normally in correlation with the biological activity desired for such heterocycles (as it happens in the case of tyrosine kinase inhibitors), the substitution pattern at C5 and C6 is responsible usually for the selectivity of one receptor with respect to other one. In this context, it is interesting to note (as it will be described later in the biological part) that the 5,6-dihydropyrido[2,3-*d*]pyrimidin-7(8*H*)-ones (**11**) with a C5-C6 single bond and the pyrido[2,3-*d*]pyrimidin-7(8*H*)-ones (**10**) with a C5-C6 double bond have been focused in very different biological targets and, consequently, the substitution pattern at C5 and C6 is quite different.

Thus, in the case of the structures **11** (C5-C6 single bond), 53.12% present at least a substituent at C5 and a CH_2_ at C6 (in most of them a carbon substituent [29,30] and, more precisely, a phenyl ring in one half of the structures [50,51]) while only 3.19% present a substituent at C6 and a CH_2_ at C5 (in this case most structures present a carbon substituent [52,53] which is a phenyl ring in also one half of them [31,42]). Finally, 20.86% of the structures do not present substituents at C5 or C6 [20,54]. These three substitution patterns cover 84.87% of the total diversity.

On the contrary, in the case of the structures **10** (C5-C6 double bond), 53.53% present only a substituent at C6 (R^5^ = H) with the following distribution referring to the total number of structures: 44.46% carbon substituent [55,56] (32.12% phenyl ring [42,57,58]), 4.70% oxygen substituent [59,60], and 1.00% nitrogen substituent [32,61]. In 7.05% of the structures there is a substituent at C5 (R^6^ = H) with the following distribution referring to the total number of structures: 4.16% carbon substituent [62,63] (0.10% phenyl ring [16,56]), 0.47% oxygen substituent [56,64], and 2.39% nitrogen substituent [64,65]. There is 7.75% of structures in which R^5^ = R^6^ = H [66,67].

These results clearly point out that 5,6-dihydropyrido[2,3-*d*]pyrimidin-7(8*H*)-ones (**11**, C5-C6 single bond) have been mainly substituted at C5 while pyrido[2,3-*d*]pyrimidin-7(8*H*)-ones (**10**, C5-C6 double bond) at C6.

### 2.3. Substitution Pattern at N8

Similarly to what has been observed for the substitution pattern at C5 and C6, the substitution pattern at N8 is clearly different between the C5-C6 single bond compounds **11** and the C5-C6 double bond compounds **10**. As it can be easily deduced from Table 3, compounds **11** have been left usually unsubstituted at N8 (R^8^ = H) while compounds **10** are usually substituted at such position, R^8^ = Me and R^8^ = cyclopentyl being the most used substituents. Once more, such pattern substitution relates to the different biological activities these two families of structures have been oriented towards.

In summary, the combinations of substituents apparently more widely explored in literature for both structures are

(a) In 14.08% of the 5,6-dihydropyrido[2,3-*d*]pyrimidin-7(8*H*)-ones (**11**): G^2^ = nitrogen substituent, G^4^ = oxygen substituent (in particular as a carbonyl group), R^5^ = phenyl group, R^6^ = H, N8 = H.
(b) In 7.84% of the pyrido[2,3-*d*]pyrimidin-7(8*H*)-ones (**10**): G^2^ = nitrogen substituent, G^4^ = H, R^5^ = H, R^6^ = phenyl group, N8 = Me.

**Figure 5:**
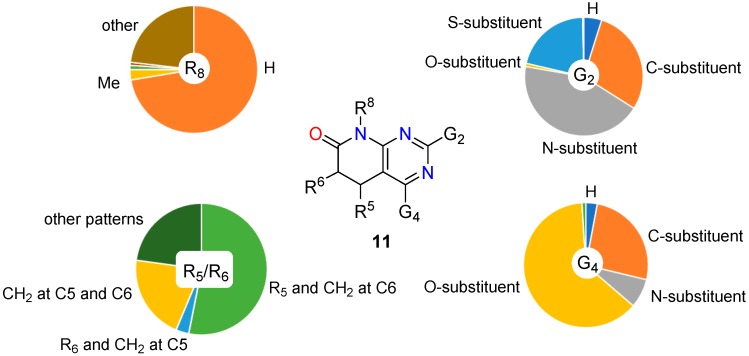
Diversity analysis of the substituents present at positions C2, C4, C5, C6, and N8 of 5,6-dihydropyrido[2,3-*d*]pyrimidin-7(8*H*)-ones (**11**) included in Scifinder.

**Figure 6:**
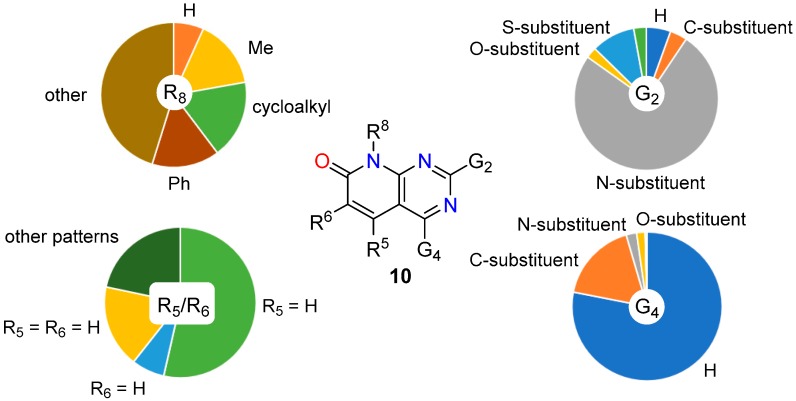
Diversity analysis of the substituents present at positions C2, C4, C5, C6, and N8 of pyrido[2,3-*d*]pyrimidin-7(8*H*)-ones (**10**) included in Scifinder.

A more visual comparison of the diversities covered by the substituents present at positions C2, C4, C5, C6, and N8 of the 5,6-dihydropyrido[2,3-*d*]pyrimidin-7(8*H*)-ones (**11**) and pyrido[2,3-*d*]pyrimidin-7(8*H*)-ones (**10**) included in *SciFinder* is included in Figure 5 and Figure 6.

Although the differences in the substitution pattern of 5,6-dihydropyrido[2,3-*d*]pyrimidin-7(8*H*)-ones (**11**) and pyrido[2,3-*d*]pyrimidin-7(8*H*)-ones (**10**) could be attributed initially to the greater or lesser synthetic accessibility of some types of substituents, the different orientation of the biological activities sought for each structure seem to be the ultimate reason for such diversity.

## 3. Synthetic Approaches to Pyrido[2,3-*d*]pyrimidin-7(8*H*)-ones

**Figure 7:**
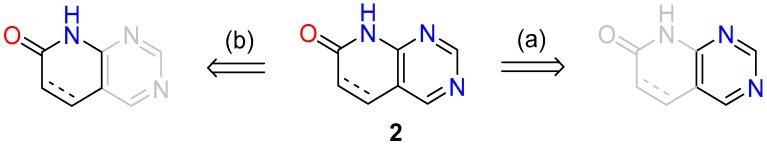
Synthetic approaches for pyrido[2,3-*d*]pyrimidin-7(8*H*)-ones (**2**): (**a**) from a preformed pyrimidine and (**b**) from a preformed pyridone.

Although for a bicyclic heterocyclic system, such as the pyrido[2,3-*d*]pyrimidin-7(8*H*)-ones (**2**), it is possible to envisage several possible synthetic approaches, in this review we concentrated on two major alternative protocols: (a) construction from a preformed pyrimidine and (b) construction from a preformed pyridone (Figure 7).

*SciFinder* offers two different ways of retrieving synthetic routes for a given general structure:

(1) Search through *Reaction Structure*: in which it is possible to draw either two general starting products and the reaction arrow not drawing the reaction product or to draw a possible general starting material and the reaction arrow followed by the structure of the reaction product. Such approaches are very convenient once the possible starting products are known.
(2) *Retrosynthetic* analysis: drawing the structure of the general final product indicating with a small arrow included in the structure editor the bonds to be broken. Such an approach is more useful when several possible synthetic approaches must be considered.

In this work, we used a combination of both methodologies to draw a picture of the synthetic approaches used for the preparation of pyrido[2,3-*d*]pyrimidin-7(8*H*)-ones (**2**).

### 3.1. Synthesis from a Preformed Pyrimidine

**Figure 8:**
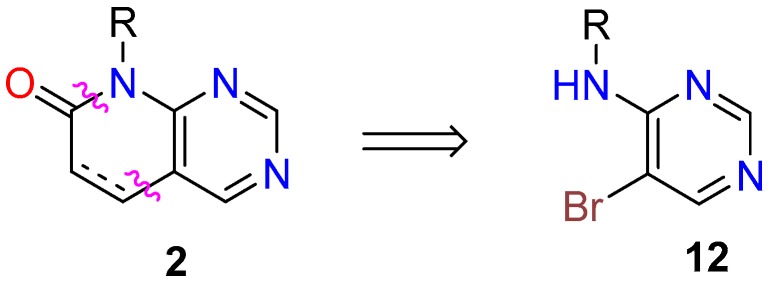
Synthetic approach for pyrido[2,3-*d*]pyrimidin-7(8*H*)-ones (**2**) from a preformed 4-amino-5-bromopyrimidine (**12**).

**Figure 9:**
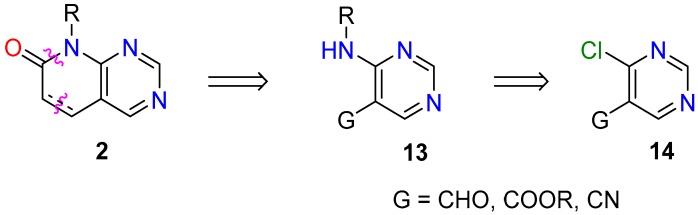
Synthetic approach for pyrido[2,3-*d*]pyrimidin-7(8*H*)-ones (**2**) from a preformed *N*-substituted pyrimidine-4-amine (**13**) bearing a carbon functional group G.

The searches carried out using the methodologies described above revealed that, in the case of preformed pyrimidines as precursors, two main approaches have been used: 

(a) In the first one, an adequately substituted 4-amino-5-bromopyrimidine (**12**) is used as the starting material. Such an approach corresponds to the disconnection of the C4a-C5 and C7-N8 bonds of the pyridopyrimidine ring (Figure 8). Compounds **12** are usually synthesized from the corresponding 5-bromo-4-chloropyridimine by reaction with the adequate amine (R-NH_2_). The search carried out in *SciFinder* indicated that there are 2563 reactions of that type which appear in 36 references, most of them patents. As for the yields of such approach, in 101 cases of the 116 fully described reactions (87.07%) they are higher than 60%.

(b) In the second one, a preformed *N*-substituted pyrimidine-4-amine (**13**) is used as starting material, which bears a carbon functional group G (CHO, COOR, or CN) at position C5 of the pyrimidine ring. Such precursor is usually formed from the corresponding 4-chloro substituted pyrimidine **14** by nucleophilic substitution with the desired amine. This synthetic approach corresponds to the disconnection of the C5-C6 and C7-N8 bonds of the pyridopyrimidine system (Figure 9). In the case of the CHO group, there are 7248 reactions included in 93 references, 3174 reactions (78 references) for the COOR group, and 115 reactions (seven references) for the CN group. Once more, most of the references are patents. In this synthetic approach, yields are higher than 60% in 840 cases of the 1476 reactions fully described (56.91%).

**Figure 10:**
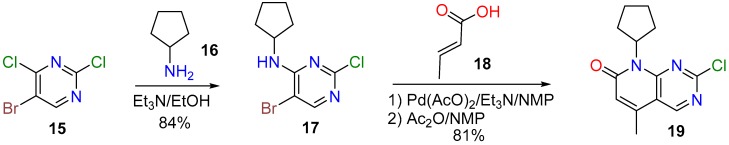
Synthesis of the palbociclib intermediate **19** from 5-bromo-2,4-dichloropyrimidine (**15**).

A nice example of the first synthetic strategy is the synthesis of **19**, an intermediate in the synthesis of palbociclib [90], starting from 5-bromo-2,4-dichloropyrimidine (**15**) which is substituted by cyclopentyl amine (**16**) to afford **17** which undergoes a palladium-catalyzed coupling with crotonic acid (**18**) followed by an intramolecular cyclization to yield the pyridopyrimidine system **19** (Figure 10).

**Figure 11:**
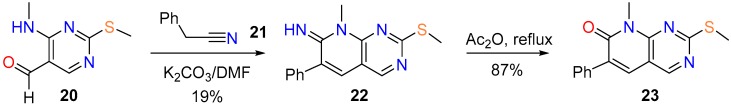
Synthesis of the pyrido[2,3-*d*]pyrimidin-7(8*H*)-one (**23**) from pyrimidine aldehyde (**20**).

Concerning the second synthetic strategy, an example of the use of a pyrimidine aldehyde for the construction of the pyridone ring is the synthesis of compound **23** [57], an intermediate for the synthesis of a series of tyrosine kinase inhibitors, starting from the aldehyde **20** which is condensed with the appropriate nitrile **21** to give the 7-iminopyridopyrimidine **22** that is subsequently transformed in the corresponding pyrido[2,3-*d*]pyrimidin-7(8*H*)-one (**23**) (Figure 11). The oxidation of the 2-methylthio substituent to a sulfone allows the introduction of arylamino substituents in such a position. In some cases, a carboxylic ester is used as a precursor of the aldehyde [91].

**Figure 12:**
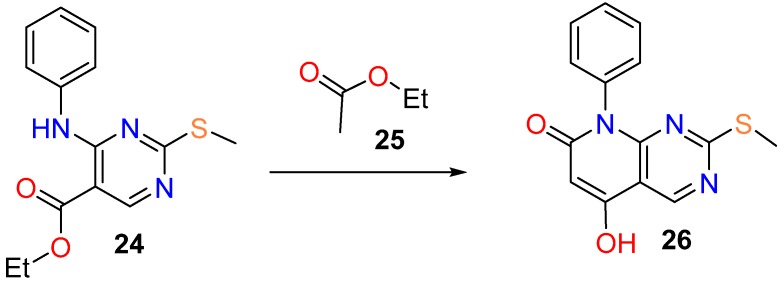
Synthesis of the 5-hydroxy substituted pyrido[2,3-*d*]pyrimidin-7(8*H*)-one (**26**) from pyrimidine ester (**24**).

An example of the direct use of a pyrimidine ester is the formation of the 5-hydroxy substituted compound **26** starting from the ester **24** upon condensation with ethyl acetate (**25**) in a multistep protocol for which the yield is not described (Figure 12) [92].

**Figure 13:**
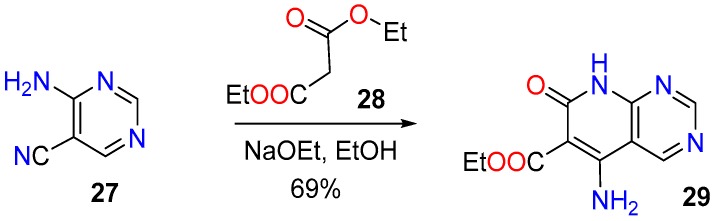
Synthesis of the 5-amino substituted pyrido[2,3-*d*]pyrimidin-7(8*H*)-one (**29**) from the nitrile substituted pyrimidine (**27**).

Finally, an example of the use of a pyrimidine nitrile as a precursor is the synthesis of the 5-amino substituted compound **29** by condensation of the nitrile substituted pyrimidine **27** with diethyl malonate (**28**) in the presence of Na/EtOH (Figure 13) [93].

The preceding synthetic protocols have two major drawbacks: on one side, it is difficult to introduce substituents in position C4 of the resulting pyridopyrimidine unless they are groups that cannot participate in the cyclization of the pyridone ring (for this reason they have mainly been used in cases that the substituent at position C4 is a hydrogen atom) and, on the other side, they afford pyrido[2,3-*d*]pyrimidin-7(8*H*)-ones (**10**) with a C5-C6 double bond.

**Figure 14:**
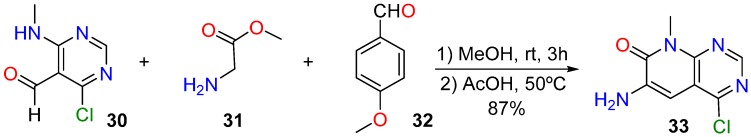
Synthesis of the 4-chloro substituted pyrido[2,3-*d*]pyrimidin-7(8*H*)-one (**33**) from the chloro substituted pyrimidine aldehyde (**30**).

The first limitation has been recently partially solved in the synthesis of the 4-chloro substituted pyridopyrimidine **33** from the chloro substituted pyrimidine aldehyde **30** (Figure 14) [33]. The presence of the 4-chloro substituent allows the ulterior substitution by nitrogen nucleophiles.

**Figure 15:**
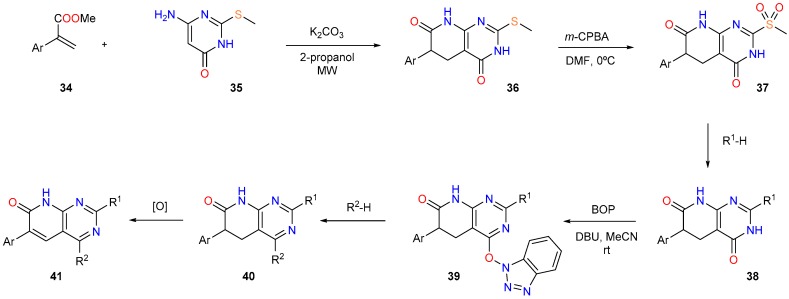
Synthesis of the substituted pyrido[2,3-*d*]pyrimidin-7(8*H*)-one (**41**) from the pyrimidinone **35**.

Our group has also contributed a complementary methodology that allows the synthesis of pyridopyrimidines with a wide range of substituents at C2 and C4 and two levels of unsaturation at C5-C6 (Figure 15) [31]. Starting from the pyrimidinone **35**, the Michael addition with a 2-aryl substituted methyl acrylate (**35**) affords the 4-oxopyridopyrimidine **36** which undergoes oxidation to the sulfone **37** that can be substituted by different nucleophiles to yield the corresponding **38**. Its reaction with benzotriazole-1-yloxytris(dimethylamino)phosphonium hexafluorophosphate (BOP) affords **39** that can be again substituted by a wide variety of nucleophiles to afford the 5,6-dihydropyrido[2,3-*d*]pyrimidin-7(8*H*)-one **40**. Finally, the oxidation of **40** affords the pyrido[2,3-*d*]pyrimidin-7(8*H*)-one **41**. The formation of the pyridopyrimidine structure proceeds normally with yields higher than 80%.

### 3.2. Synthesis from a Preformed Pyridone

**Figure 16:**
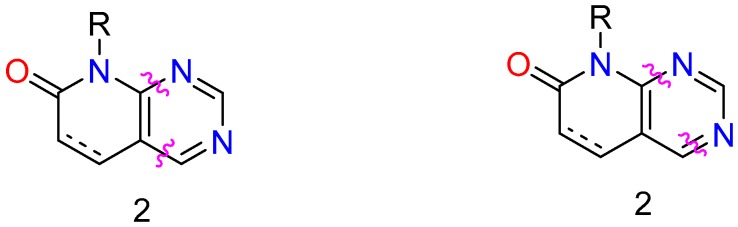
Synthetic approaches for pyrido[2,3-*d*]pyrimidin-7(8*H*)-ones (**2**) from a preformed pyridone.

In this second synthetic approach, we considered two possible disconnections: (a) in between C8a-N1 and C4-C4a and (b) in between C8a-N1 and N3-C4 (Figure 16).

**Figure 17:**
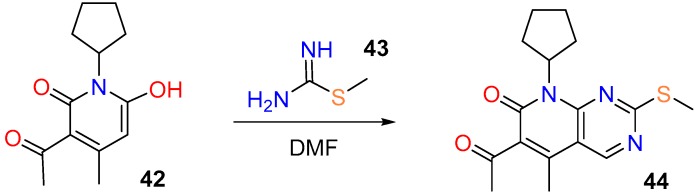
Synthesis of the substituted pyrido[2,3-*d*]pyrimidin-7(8*H*)-one (**44**) from the pyridone **42**.

Only one example of the first kind of disconnection was found in *SciFinder*, the synthesis of compound **44** [94], an intermediate in the synthesis of pobosaibu, from pyridone **42** upon treatment with *S*-methylisothiourea (**43**) and DMF (*N,N*-Dimethylformamide) which provides the C4 carbon atom (yield not available) (Figure 17).

**Figure 18:**
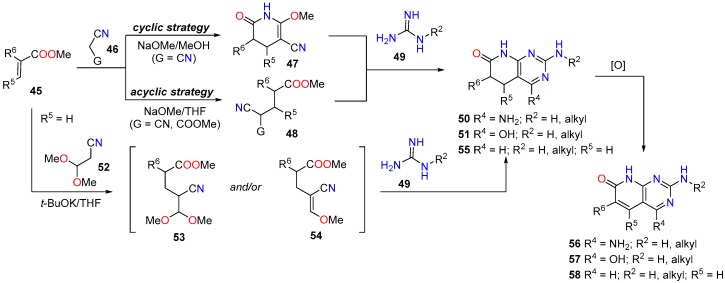
Synthesis of 5,6-dihydropyrido[2,3-*d*]pyrimidin-7-(8*H*)-ones and pyrido[2,3-*d*]pyrimidin-7-(8*H*)-ones from α,β-unsaturated esters (**45**).

However, there are many examples of the second disconnection option shown in Figure 16. In this context, our group has a broad experience in the synthesis of 5,6-dihydropyrido[2,3-*d*]pyrimidin-7-(8*H*)-ones (**50**; R^4^ = NH_2_) and (**51**; R^4^ = OH) from α,β-unsaturated esters (**45**) (Figure 18). Thus, in the so-called *cyclic strategy* 2-methoxy-6-oxo-1,4,5,6-tetrahydropyridine-3-carbonitriles (**47**) are obtained by reaction of an α,β-unsaturated ester (**45**) and malononitrile (**46**, G = CN) in NaOMe/MeOH (usually yields are above 60% although it depends on the nature and position of the substituents at C5 and C6 [95,96]). Treatment of pyridones **47** with guanidine systems (**49**, R^2^ = H, alkyl) affords 4-amino-pyrido[2,3-*d*]pyrimidines (**50**, R^4^ = NH_2_) [95,96]. On the other hand, we described an *acyclic* variation of the above protocol for the synthesis of pyridopyrimidines (**50**, R^4^ = NH_2_) based on the isolation of the corresponding Michael adduct (**48**, G = CN) and later cyclization with a guanidine **49** [24]. Similarly, 4-oxopyrido[2,3-*d*]pyrimidines (here depicted as the hydroxyl tautomer **51**, R^3^ = OH) are obtained by treating intermediates (**48**, G = CO_2_Me), the result of a Michael addition between **45** and methyl cyanoacetate (**46**, G = CO_2_Me), with guanidines **49** [97]. Such *acyclic* protocol was amenable to a multicomponent microwave-assisted cyclocondensation to afford compounds **50** and **51** via Michael adducts **48** [50,98]. We also achieved 4-unsubstituted 5,6-dihydropyrido[2,3-*d*]pyrimidines (**55**; R^4^ = H) through the Michael addition of 2-aryl substituted acrylates (**45**; R^6^ = aryl, R^5^ = H) and 3,3-dimethoxypropanenitrile (**52**) which leads, depending on the reaction temperature (60 or −78 °C, respectively), to a 4-methoxymethylene substituted 4-cyanobutyric ester (**54**) or to a 4-dimethoxymethyl 4-cyanobutyric ester (**53**). These compounds are subsequently converted to the desired 4-unsubstituted compound (**55**; R^4^ = H) upon treatment with a guanidine carbonate **49** under microwave irradiation [99]. We completed our approach to totally dehydrogenated pyrido[2,3-*d*]pyrimidin-7(8*H*)-ones (**56**–**58**) and (**17**; R^4^ = H) by using several oxidation protocols [16]. The construction of the pyridopyrimidine structure from pyridone **47** usually proceeds with yields higher than 70% but is mainly dependent on the nature and position of the substituents present in the pyridone ring.

**Figure 19:**
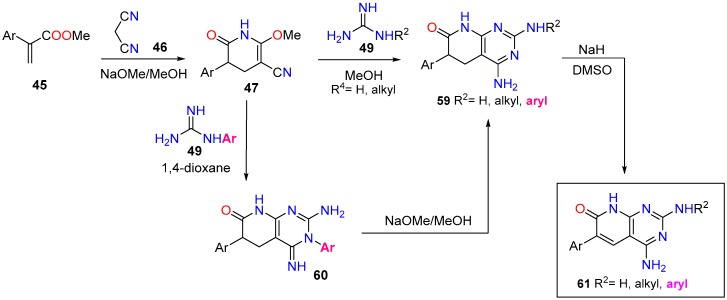
Synthesis of 6-aryl-2-arylamino substituted 4-amino-5,6-dihydropyrido[2,3-*d*]pyrimidin-7(8*H*)-ones (**59**) from α,β-unsaturated esters (**45**).

Our interest in tyrosine kinase inhibitors, led us to develop a methodology for the synthesis of 6-aryl-2-arylamino substituted 4-amino-5,6-dihydropyrido[2,3-*d*]pyrimidin-7(8*H*)-ones (**59**, R^2^ = aryl) via the corresponding 3-aryl substituted pyridopyrimidines **60**, formed upon treatment of pyridones **47** with an arylsubstituted guanidine **49** in dioxane, which underwent the Dimroth rearrangement to the desired 4-aminopyridopyrimidines (**59**, R^2^ = aryl) with NaOMe/MeOH. The overall yields of such a two-step protocol are in general higher than the direct reaction between pyridones **47** and an arylsubstituted guanidine **49** (Figure 19) [28].

Outside of these two main methodologies, the rest of the literature concerning the synthesis of pyrido[2,3-*d*]pyrimidine-7(8*H*)-ones is devoted to the manipulation of the substituents present in the bicyclic ring, mainly at C2 and C4, by using nucleophilic substitutions of halogen atoms at such positions by nitrogen or oxygen nucleophiles. Thus, there are more than 2000 examples of the substitution of a 2-chloropyridopyrimidine by nitrogen nucleophiles in good yields [62] but fewer of the substitution of a 2-bromo substituted compound [100]. Similarly, the substitution of the corresponding 4-chloropyrimidine by nitrogen nucleophiles appears in more than 1800 examples [33] but the same reaction carried out with 4-bromo derivatives only appears in a reference of our research group [52]. Even more frequently, the introduction of nitrogen or oxygen substituents at position C2 is carried out in a two steps protocol in which a methyl thioether is oxidized to the corresponding methyl sulfonyl group (usually with m-CPBA) and subsequently substituted by the nitrogen or oxygen nucleophile. On example of such two steps protocol, used in more than 2000 reactions in the literature, is depicted in Figure 15.

On the other hand, two miscellaneous protocols for the formation of the pyrido[2,3-*d*]pyrimidine-7(8*H*)-one scaffold include a Diels-Alder reaction between diethyl acetylenedicarboxylate and a 6-amino-4-oxopyrimidine ring [101] and the photochemical cyclization of *N*-(pyrimidin-4-yl)methacrylamide [14], however, both methodologies proceed in very low yields (1% and 21% respectively).

## 4. Biomedical Applications of Pyrido[2,3-*d*]pyrimidin-7(8*H*)-ones

*SciFinder* is a very useful tool when one is interested in the biological activity or uses of a single compound but renders it difficult to retrieve biological information for a family of compounds. Certainly, it is possible to retrieve all the references including biological data for a set of structures (*Biological Study*) but it only retrieves the references that later must be examined one by one (in the case of the 14,448 structures with a C5-C6 double bond reduces the number by 10%). For this reason, we used the tool *Index Term* to obtain a list, ordered by frequency, of the indexed terms (keywords) that appear in the references in order to get a general picture of the biological activities of 5,6-dihydropyrido[2,3-*d*]pyrimidin-7(8*H*)-ones (**11**) and pyrido[2,3-*d*]pyrimidin-7(8*H*)-ones (**10**). Table 4 shows the 10 first *index terms* obtained for compounds **11** (C5-C6 single bond) and **10** (C5-C6 double bond).

The simple inspection of Table 4 clearly indicates that the intended uses of both families of compounds are totally different. On one side, 5,6-dihydropyrido[2,3-*d*]pyrimidin-7(8*H*)-ones (**11**) have been applied for cardiovascular diseases: antihypertensive agents (as angiotensin II receptor antagonists), antidiabetics, etc., although also as antitumor agents. In particular, there are circa 150 references including the index term antihypertensives [102,103]. On the other side, pyrido[2,3-*d*]pyrimidin-7(8*H*)-ones (**10**) have been focused on antitumor agents: mammary neoplasms, neoplasms, signal transduction, etc., in particular as tyrosine kinase inhibitors (around 430 references) [104,105].

**Figure 20:**
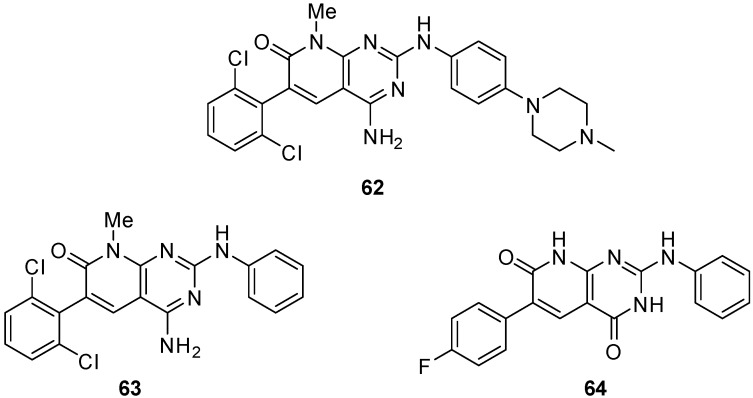
Pyrido[2,3-*d*]pyrimidin-7(8*H*)-ones with nM biological activities as BCR kinase inhibitor (**62**), DDR2 inhibitor (**63**), and HCV NS5B polymerase inhibitor (**64**).

In this context, our group has described in the past years several 4-amino and 4-oxo substituted pyrido[2,3-*d*]pyrimidin-7(8*H*)-ones (R^4^ = NH_2_, OH) (Figure 18 and Figure 19 with up to five diversity centers which, contrary to compounds bearing R^4^ = H, render these compounds, in general, nontoxic for normal cells. Consequently, an adequate decoration of these structures has allowed us to describe compounds with activities in the nanomolar range as BCR kinase (Breakpoint Cluster Region protein) inhibitors for B lymphoid malignancies (compound **62**) [42], DDR2 (Discoidin Domain-containing Receptor 2) inhibitors for treatment of lung cancer (compound **63**) [106], and even as HCV (Hepatitis C Virus) inhibitors (compound **64**) [31], and others (Figure 20). In fact, we are convinced that an adequate decoration of pyrido[2,3-*d*]pyrimidin-7(8*H*)-one scaffolds should allow targeting very diverse biological receptors such, in our case, tyrosine kinases and HCV NS5B polymerase.

Reaching this point, it would be highly interesting to know how many 5,6-dihydropyrido[2,3-*d*]pyrimidin-7(8*H*)-ones (**11**) and pyrido[2,3-*d*]pyrimidin-7(8*H*)-ones (**10**) have reached the market. *SciFinder* is not the right tool to find this information since it does not incorporate, at least with clarity, commercial information or the stage of development of a drug candidate. Consequently, we used a second database called *Drugbank* [107] (https://www.drugbank.ca/) for searching the market situation of compounds **10** and **11**.

**Figure 21:**
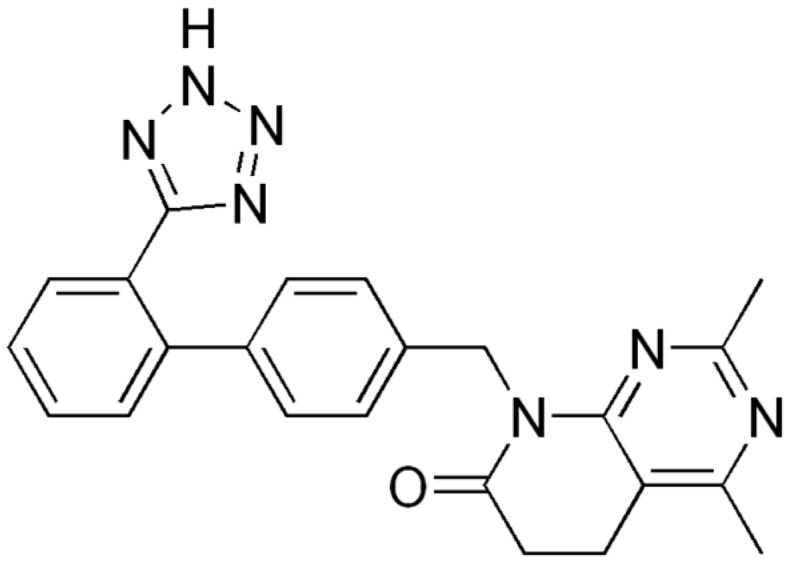
Tasosartan (**65**).

The search showed that in the case of 5,6-dihydropyrido[2,3-*d*]pyrimidin-7(8*H*)-ones (**11**) a single compound is included in the database. Tasosartan (**65**, CAS 145733-36-4, Figure 21), patented by American Home Products Corporation and developed by Wyeth as an angiotensin II receptor antagonist, did not reach the market since it was withdrawn by the manufacturer during the Clinical Phase III due to the detection of elevated transaminase levels (as a sign of possible liver toxicity) in a high number of participants in the study.

On the other hand, in the case of pyrido[2,3-*d*]pyrimidin-7(8*H*)-ones (**10**), there are eight structures in various stages of development. Two of them are classified as *Experimental*, a category that according to *Drugbank* corresponds to potential drugs in the Discovery Phase. Five more in the *Investigational* category, which means that for these compounds an IND (Investigative New Drug) application has been submitted to the FDA (Food and Drug Administration) indicating that they have initiated Clinical Phases. Finally, there is a single compound approved by the FDA, the already mentioned palbociclib (**8**, CAS 571190-30-2, Figure 3). All these structures have a nitrogen substituent at C2 and hydrogen at C4 in line with the structural searches carried out in this work.

Palbociclib was patented by Warner-Lambert in 2003 [108]. Palbociclib [109,110] is a pyrido[2,3-*d*]pyrimidin-7(8*H*)-one that acts in the cell cycle machinery [111]. It is a second inhibitor of CD4 kinase and was selected from a group of pyridopyrimidines due to its favorable physical and pharmaceutical properties. Palbociclib was developed by Pfizer Inc after the discovery that identified cyclin-dependent kinases as key regulators of cell growth. It was approved by the FDA in March 2015 for the treatment of breast cancer positive for HR, advanced HER2-negative, or metastatic. The indications were updated in April 2019 to include male patients based on the results of post-marketing reports that demonstrate clinical safety and efficacy.

**Figure 22:**
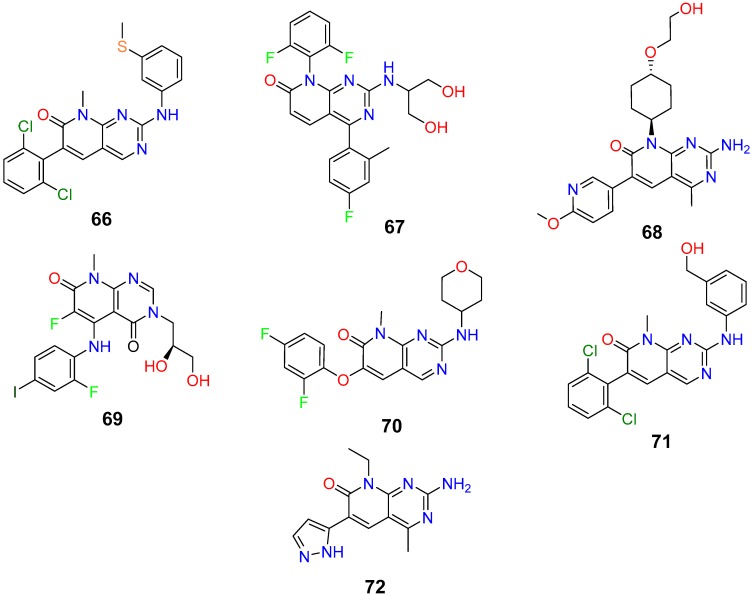
Structures of pyrido[2,3-*d*]pyrimidin-7(8*H*)-ones (**10**) included in Drugbank which are in different phases of development.

As for the rest of pyrido[2,3-*d*]pyrimidin-7(8*H*)-ones (**10**) under development (Figure 22), it is interesting to note that the list includes some of the most important pharmaceutical companies (Takeda, Pfizer, SmithKline Beecham, etc.) thus confirming the interest of structures **10** as scaffolds for the development of new drugs.

Compound **66** (CAS 260415-63-2) was patented by the Scripps Research Institute in 2001 [112] as an Abl1/Src kinase inhibitor mainly oriented to leukemia treatment although more recent papers and patents seem to address this compound also to neuromuscular disorders. To the best of our knowledge, this compound has not yet reached the market.

Dilmapimod (**67**, CAS 444606-18-2) is a p38 MAP-kinase inhibitor with potential uses in rheumatoid arthritis that was patented by SmithKline Beecham Corporation (GSK-681323) in 2001 [113] and has been involved in clinical trials for inflammation, neuropathic pain, and heart diseases but was finally discontinued due to liver toxicity. 

Compound **68** (CAS 1013101-36-4) was patented by Pfizer in 2007 [114] as a 1 phosphatidylinositol 3 kinase inhibitor and MTOR protein inhibitor. It reached Phase II clinical trial for endometrial cancer treatment but was discontinued in 2012. N68 has also been involved in clinical trials for early breast cancer (Phase 2), and advanced breast cancer (Phase 1b). More than 180 publications including many patents, some of them very recent, seem to indicate that there is still an interest of Pfizer for such compounds.

TAK-733 (**69**, CAS 1035555-63-5) is a compound developed and patented by Takeda Pharmaceutical Company in 2007 [115] as an inhibitor of MEK1 and MEK2 (MEK1/2) that was tested in clinical trials for advanced metastatic melanoma and advanced nonhematologic malignancies. TAK-733 was tested against non-small cell lung cancer reaching Phase I but was discontinued in 2015.

Compound **70** (CAS 449811-92-1), patented by Hoffmann-La Roche (R-1487) in 2002 [116] for the treatment of p38 mediated disorders, was investigated for use/treatment in rheumatoid arthritis but was discontinued in 2004.

Compound **71** (CAS 185039-91-2) was patented by Warner-Lambert Company in 1996 [117]. Warner-Lambert was very active in the field of pyrido[2,3-*d*]pyrimidin-7(8*H*)-ones (**10**) in the late 90s. Such company was bought by Pfizer in 2000 and this fact seems to have stopped the development of such compound.

Finally, Voxtalisib (**72**, CAS 934493-76-2) was patented by Exelis (XL-765) in 2006 [118] as an inhibitor of phosphatidylinositol 3 kinase (PI3K) and mammalian target of rapamycin (mTOR) kinases in the PI3K/mTOR signaling pathway. **72** was licensed to Sanofi-Aventis (SAR-245409) in 2009 in a deal for the development PI3K inhibitors with an upfront payment of $140 million and guaranteed research funding. In 2018, Voxtalisib was discontinued in Phase I/II for solid tumors (breast and ovarian cancer) both as monotherapy and combined therapy.

## 5. Conclusions

In this paper, we reviewed the substitution patterns of 5,6-dihydropyrido[2,3-*d*]pyrimidin-7(8*H*)-ones (**11**) and pyrido[2,3-*d*]pyrimidin-7(8*H*)-ones (**10**) establishing the kind of substituents mainly used at positions C2, C4, C5, C6, and N8 of such systems. For compounds **11**, we established that the most used combination of substituents is G^2^ = nitrogen substituent, G^4^ = oxygen substituent (in particular as a carbonyl group), R^5^ = phenyl group, R^6^ = H, N8 = H. In the case of compounds **10**, it is G^2^ = nitrogen substituent, G^4^ = H, R^5^ = H, R^6^ = phenyl group, N8 = methyl.

We established the main synthetic strategies for the synthesis of such compounds starting from a preformed pyrimidine or pyridone and demonstrated that compounds **11** have been mainly used in the area of cardiovascular diseases while compounds **10** have been focused in antitumor agents, mainly as tyrosine kinase inhibitors. 

## Figures and Tables

**Table 1:**
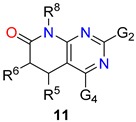
Substitution pattern at C2 and C4 of 5,6-dihydropyrido[2,3-*d*]pyrimidin-7(8*H*)-ones (**11**) with a C5-C6 single bond.

Substituent	G^2^		G^4^	
	Structures (%)	References	Structures (%)	References
H	4.87	22 [14,15]	2.96	48 [16,17]
C	29.10	335 [18,19]	25.80	317 [20,21]
N	43.78	80 [22,23]	7.53	57 [24,25]
O	0.88	25 [26,27]	62.70	317 [28,29]
S	21.02	18 [30,31]	0	-

**Table 2:**
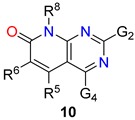
Substitution pattern at C2 and C4 of pyrido[2,3-*d*]pyrimidin-7(8*H*)-ones (**10**) with a C5-C6 double bond.

Substituent	G^2^		G^4^	
	Structures (%)	References	Structures (%)	References
H	5.48	108 [32,33]	78.10	1946 [34,35]
C	3.86	93 [36,37]	17.42	447 [38,39]
N	75.55	2220 [40,41]	2.25	92 [42,43]
O	2.45	98 [44,45]	1.82	93 [27,46]
S	9.84	243 [47,48]	0.05	3 [37,49]

**Table 3:** Substitution pattern at N8 of 5,6-dihydropyrido[2,3-*d*]pyrimidin-7(8*H*)-ones (**11**) with a C5-C6 single bond and the pyrido[2,3-*d*]pyrimidin-7(8*H*)-ones (**10**) with a C5-C6 double bond.

R^8^	Structures 11 (%)	References	Structures 10 (%)	References
H	72.37	[29,31]	6.71	[44,68]
Me	2.62	[42,69]	15.48	[70,71]
Et	0.11	[72,73]	7.03	[74,75]
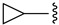	0.29	-	0.84	[76,77]
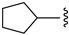	0.74	[78,79]	15.95	[80,81]
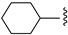	0.04	[73]	0.78	[82,83]
Ph	0.77	[84,85]	15.01	[62,86]
OR	-	-	5.52	[87,88]
NR	0.03	[89]	0.73	[36,76]

**Table 4:** *Index terms* in *SciFinder* for compounds **10** and **11**.

Compounds 11		Compounds 10	
Index Term	Frequency	Index Term	Frequency
Human	170	Human	1300
Antihypertensives	150	Antitumor agents	1028
Hypertension	120	Mammary gland neoplasm	535
Combination chemotherapy	96	Neoplasm	523
Angiotensin II receptor antagonists	95	Combination chemotherapy	503
Drug delivery systems	90	Piperazines	385
Cardiovascular agents	66	Pyridines	380
Diabetes mellitus	62	Signal transduction	365
Heart failure	61	Cell proliferation	346
Antitumor agents	61	Proteins	345
